# Association between PET–CT accumulation in the hypothalamic/pituitary regions and neuron-specific enolase/primary tumor in limited-stage small cell lung cancer: a case-controlled retrospective study

**DOI:** 10.1186/s41824-024-00190-z

**Published:** 2024-02-05

**Authors:** Yukinori Okada, Tatsuhiko Zama, Tomohiro Itonaga, Ryuji Mikami, Mitsuru Okubo, Shinji Sugahara, Motoki Nakai, Koichiro Abe, Mana Yoshimura, Kazuhiro Saito

**Affiliations:** Department of Radiology, Tokyo Medical University, Tokyo, Japan

**Keywords:** Positron emission tomography–computed tomography, Neuron-specific enolase, Standardized uptake value, Small cell lung cancer, Hypothalamus/pituitary

## Abstract

**Background:**

Research on the relationship between neuron-specific enolase (NSE) levels and normal organs, particularly the central nervous system, in small cell lung cancer is limited. Therefore, this study aimed to investigate the relationship between positron emission tomography–computed tomography (PET–CT) accumulation at hypothalamic/pituitary regions, tumor activity, and NSE level in limited-stage small cell lung cancer. We retrospectively analyzed patients who were diagnosed with limited-stage small cell lung cancer at Tokyo Medical University Hospital between July 1, 2019, and May 31, 2023, and were treated with chemoradiotherapy or radiotherapy. Leukocytes, erythrocytes, hemoglobin, platelets, total protein, albumin, NSE, and carcinoembryonic antigen were measured in blood samples obtained before treatment initiation. The maximum standardized uptake value (SUVmax), volume, and total lesion glycolysis (TLG) of each hypothalamic /pituitary region, primary tumor, and lymph node metastases were extracted from PET–CT images. The total tumor volume (primary tumor volume plus lymph node metastases volume) and total TLG (primary tumor TLG plus lymph node metastases TLG) were calculated.

**Results:**

This study included 19 patients (mean age, 70.1 ± 8.8 years; 13 men and 6 women); the pathology in all patients was small cell lung cancer. Patients were classified into two groups according to the NSE reference value (16.3 ng/mL): six patients having NSE level below the reference value and 13 having NSE level above the reference value. The SUVmax in the hypothalamic/pituitary region was 2.95 in the NSE < 16.3 ng/mL group and 4.10 in the NSE > 16.3 ng/mL group, with a statistically significant difference (*p* = 0.03). The total tumor volume was 17.8 mL in the NSE < 16.3 ng/mL group and 98.9 mL in the NSE > 16.3 ng/mL group, with a statistically significant difference (*p* < 0.01). A correlation coefficient of *r* = 0.458 (*p* = 0.0486) was observed between SUVmax in the hypothalamus/pituitary and NSE level. A correlation coefficient of *r* = 0.647 (*p* < 0.01) was also observed between total tumor volume and NSE level. Finally, a correlation coefficient of *r* = 0.53 (*p* = 0.01) was observed between hypothalamic/pituitary TLG and primary tumor TLG.

**Conclusions:**

The findings demonstrated a correlation between hypothalamic/pituitary activity and tumor activity, suggesting the prognostic significance of NSE.

## Background

Small cell lung cancer is a type of malignant tumor that accounts for approximately 15% of all primary lung cancers (Rudin et al. 2021). In addition to the TNM classification, small cell lung cancer is classified into limited and extensive stages, indicating that radical irradiation is feasible or difficult, respectively. Chemoradiation and chemotherapy are the standard treatments for limited-stage and extensive-stage disease, respectively (Rudin et al. 2021). However, the prognosis of small cell lung cancer is poor, with a reported 5-year survival rate of 25% for limited-stage disease and 5% for extensive-stage disease (Rudin et al. 2021).

Small cell lung cancer is diagnosed using bronchoscopy, with biopsy performed for pathological confirmation. Staging is performed using various methods, including whole-body computed tomography (CT), positron emission tomography–CT (PET–CT), and head magnetic resonance imaging (MRI). Moreover, neuron-specific enolase (NSE) and pro-gastrin-releasing peptide (ProGRP) serve as tumor markers.

NSE is an isoenzyme of a glycolytic enzyme and has been reported to be characteristic of neuronal and neuroendocrine tumors (Isgrò et al. 2015). A study of 263 cases of small cell lung cancer reported that 79% of patients had NSE levels above the reference value (Quoix et al. 2000). A study comparing 102 cases of small cell lung cancer and 60 cases of benign lung tumors found that M2-pyruvate kinase, NSE, and ProGRP had sensitivities of 82.35%, 60.78%, and 77.45%, respectively, and specificities of 91.11%, 81.11%, and 86.67%, respectively, for small cell lung cancer (Li et al. 2023). NSE is not specific to small cell lung cancer; however, it is considered effective for evaluating the pathology and therapeutic effects of treatment. NSE values and tumor spread have been reported to be correlated (Isgrò et al. 2015). In addition, a study of 64 cases of limited-stage small cell lung cancer found that the increases in ProGRP, NSE, cytokeratin fraction 21–1, and lactate dehydrogenase (LDH) levels were 79.7%, 57.8%, 23.4%, and 12.5%, respectively, with ProGRP level ≥ 410 ng/mL and NSE level ≥ 46 µg/L being associated with poor overall survival (Wójcik et al. 2008). Therefore, NSE is considered effective for the diagnosis and prognosis of small cell lung cancer.

18F-fluorodeoxyglucose (^18^F-FDG), a glucose-like substance, is used in the diagnostic imaging of malignant tumors. A previous review has reported that ^18^F-FDG PET–CT is superior to conventional examination methods for staging small cell lung cancer (Ruben and Ball 2012). A study of 118 cases of small cell lung cancer (50 limited-stage and 68 extensive-stage cases) found that the odds ratio for the maximum standardized uptake value (SUVmax) of the primary tumor was 1.24 in the limited stage and that for metabolic tumor volume (MTV) was 1.001 in the extensive stage. Total lesion glycolysis (TLG) had an odds ratio of 1.0003. Therefore, these have been reported to be statistically significant prognostic factors (Choi et al. 2020). In addition, a study of 21 cases of recurrent small cell lung cancer reported no correlation between tumor MTV, TLG, and NSE in patients with NSE levels within reference value however, NSE, TLG, and MTV showed high correlation coefficients of 0.788–0.866 in the group with NSE levels above reference value (Shi et al. 2015). Both NSE level and PET–CT are effective for diagnosing small cell lung cancer and evaluating therapeutic effects. However, NSE is present in cancer types beyond small cell lung cancer. NSE has been found in neurons and accounts for 0.4–2.2% of brain proteins; however, it is not found in glial cells (Isgrò et al. 2015; Marangos and Schmechel 1987). Furthermore, NSE level is reported to increase during brain death, i.e., hypoxia (Isgrò et al. 2015). However, only few reports have examined the relationship between NSE levels and normal organs, particularly the central nervous system, in small cell lung cancer. Moreover, an unpublished study reported that small cell lung cancer uses the function of the pituitary gland, particularly of the anterior pituitary gland, to proliferate, subsequently worsening the patient's prognosis (KAKEN 2017).

This study aimed to investigate the relationship between normal organs, particularly the hypothalamic/pituitary regions, and tumor activity in small cell lung cancer.

## Methods

### Research design

This was a case-controlled, retrospective study conducted at a single institution.

### Patient selection

Patients aged between 20 and 100 years, diagnosed with limited-stage small cell lung cancer based on pathology, tumor markers, imaging findings, and clinical course at Tokyo Medical University Hospital between July 1, 2019, and May 31, 2023, and treated with chemoradiotherapy or radiotherapy were screened for inclusion.

The exclusion criteria were set as follows: participants (1) with active multiple cancers at the time of diagnosis of small cell lung cancer; (2) with a high possibility of multiple cancers diagnosed based on clinical history and imaging, among others, at the time of diagnosis of small cell lung cancer; (3) receiving treatment for other active cancers at the time of diagnosis of small cell lung cancer; (4) involved in other clinical trials or research; and (5) that did not consent to data usage for this study. However, patients with a history of other cancers undergoing follow-up without treatment and those with recurrence or metastasis of other cancers that could not be detected by PET–CT, CT, or MRI were included. Similarly, patients who were previously included in this study and were subsequently involved in other studies in different departments were included.

### Blood sample findings

Data on leukocytes, erythrocytes, hemoglobin, platelets, total protein, albumin, NSE, and carcinoembryonic antigen (CEA) were extracted from blood samples obtained before the initiation of treatment for localized small cell lung cancer.

### PET–CT

^18^F-FDG (Nihon Medi-Physics Co, Ltd., Tokyo, Japan) was administered intravenously according to the body weight of each patient at a dose of 3.7 MBq/kg, and PET–CT (Discovery MI: GE Healthcare, Hino, Japan) was performed after approximately 60 min of rest. This PET–CT (Discovery MI) used a semiconductor and a Silicon Photomultiplier (SiPM). Q.Clear (use block sequential regularized expectation maximization) was used for image reconstruction.

### Analysis of PET–CT images

We analyzed the PET–CT images used for initial staging. PET–CT was performed by a radiologist (OY) who is a radiation oncologist, a nuclear medicine specialist, and a PET nuclear medicine-certified physician with experience in PET–CT-related research (Sawaragi et al. 2023; Okumura et al. 2022). The PET–CT images were visually evaluated by the same radiologist (OY). We imported the PET–CT images into MIM Maestro (Euro Meditech Co. Ltd, Tokyo, Japan/MIM Software Co. Ltd, Cleveland, OH, USA) and input the target primary tumor, lymph node metastases tumor, and hypothalamus/ pituitary regions. Maximum standardized uptake value (SUVmax), tumor volume (mL), and TLG were calculated by MIM Maestro. We used the following formula to calculate TLG: TLG = SUVmean (mean standard uptake value) × volume. We used primary tumor SUVmean and lymph node metastases SUVmean, rather than the total tumor SUVmean, to calculate total tumor TLG. Moreover, we evaluated the maximum diameter of the primary tumor. Furthermore, we visually confirmed the absence of brain metastases and obvious abnormalities in the hypothalamic and pituitary regions by contrast-enhanced CT or MRI, which was performed at the first visit for staging.

### Statistical analysis

EZR software (Jichi Medical University Omiya Medical Center, Tochigi, Japan) was used for statistical analysis (Kanda 2013). The Mann–Whitney U and Fisher’s exact tests were used for comparison between the two groups. Cutoff values were calculated using receiver operating characteristic (ROC) analysis. Spearman's rank correlation coefficient was used to calculate the correlation coefficient. Statistical significance was set at *p*-value < 0.05.

## Results

### Study patients

A total of 19 patients were included. The mean age was 70.1 ± 8.8 years, (range 54–84 year). Thirteen patients were men, and 6 patients were women. Only one patient refused data usage and was excluded. The pathology of all the cases was small cell lung cancer, and all cases were in the limited stage. Furthermore, one case had concurrent adrenocortical adenoma treated with corticosteroids, and one case had concurrent human immunodeficiency virus infection controlled with drug therapy.

### Blood sample findings

The blood sample findings were as follows: white blood cell count, 7.34 ± 1.56 × 10^3^/µL; red blood cell count, 4.26 ± 0.68 × 10^6^/µL; hemoglobin level, 13.0 ± 1.8 g/dL, platelet count, 288.5 ± 138.6 × 103/µL; total protein level, 7.1 ± 0.65 g/dL; albumin level, 3.96 ± 0.50 g/dL; NSE level, 29.5 ± 16.7 ng/mL; and CEA level, 6.36 ± 5.85 ng/mL.

### PET–CT and head CT/MRI findings

**Fig. 1:**
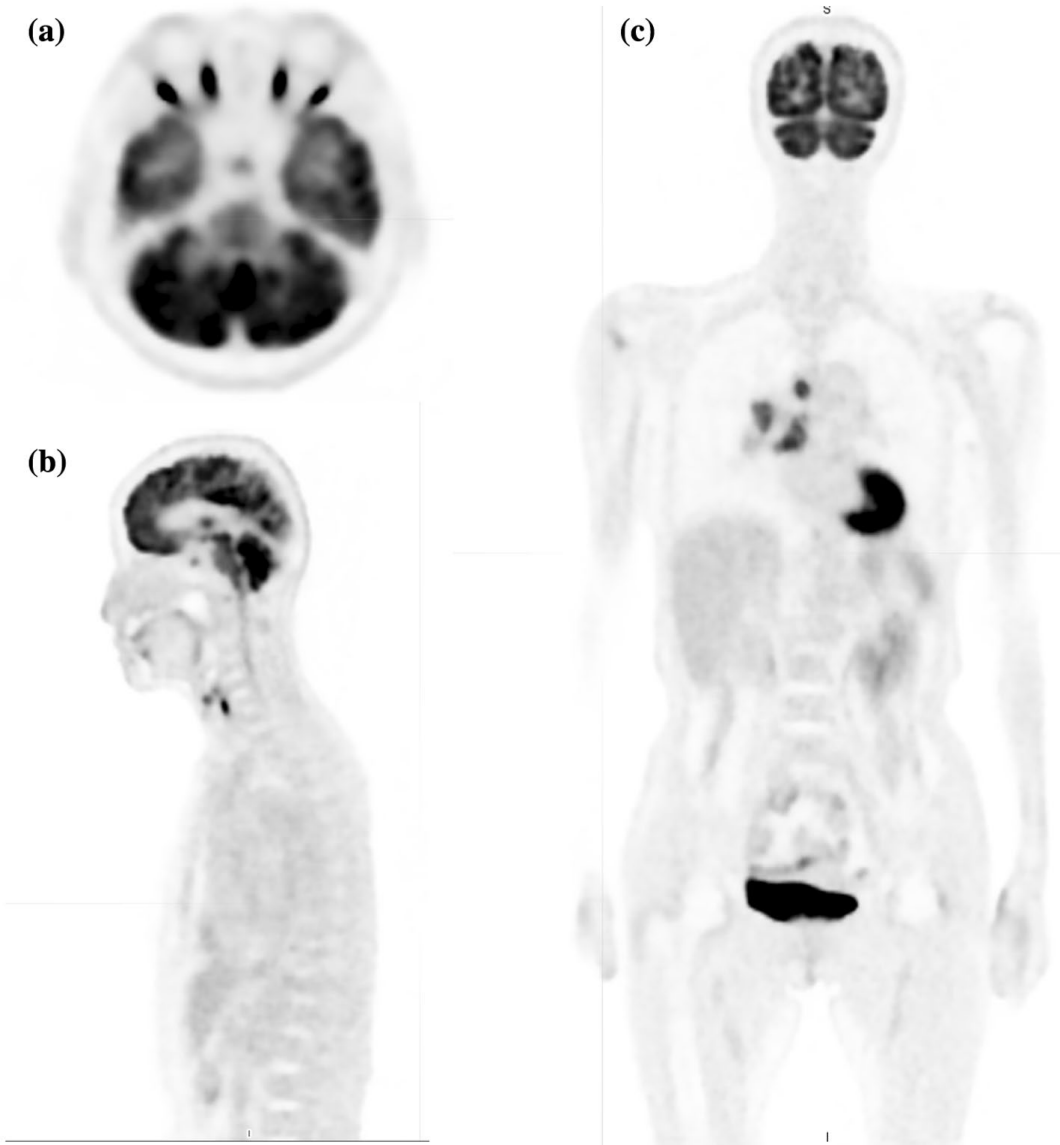
The PET–CT images (hypothalamic/pituitary region and whole body) were shown

The mean blood glucose level was 112.9 ± 14.9 mg/dL. The mean ^18^F-FDG dose was 235.1 ± 39.0 MBq. One patient had the primary tumor and lymph node metastases lumped together at the mediastinum; therefore, we classified the condition in these patients as a primary node metastases pattern and calculated the SUVmax, TLG, and volume for only the primary tumor. The SUVmax in the hypothalamic/pituitary region was 3.47 ± 0.80, the volume of the hypothalamic/pituitary region was 0.52 ± 0.13 mL, and the TLG of the hypothalamic/pituitary region was 1.33 ± 0.33 SUV × mL. The SUVmax, volume, and TLG of the primary tumor were 13.3 ± 3.5, 42.0 ± 39.7 mL, and 314.9 ± 348.0 SUV × mL, respectively. The maximum diameter of the primary tumor was 40.2 ± 19.0 mm. The SUVmax, volume, and TLG of the lymph node metastases were 11.6 ± 4.4, 29.6 ± 33.0 mL, and 208.6 ± 263.0 SUV × mL, respectively. The total tumor volume (primary tumor volume plus lymph node metastases volume) was 70.1 ± 53.0 mL, and the total tumor TLG (primary tumor TLG plus lymph node metastases TLG) was 512.6 ± 461.4 SUV × mL. No clear abnormalities were found in the pituitary region and brain metastases in 18 cases of head MRI with contrast and one case of head CT with contrast (the patient was shortly post-surgery and was unable to undergo MRI). These findings are tabulated in Table 1. Moreover, the PET–CT images (hypothalamic/pituitary region and whole body) are shown in Fig. 1a, b, and c (71-year-old women).

**Table 1:** Cases, blood sample findings, and PET–CT findings

Factors	Results
*Patient background*	
Cases	19 Cases
Age	70.1 ± 8.8 years
Sex	13 Male, 6 female
*Blood samples*	
White blood cells	7.34 ± 1.56 × 10^3^/µL
Red blood cells	4.26 ± 0.68 × 10^6^/µL
Hemoglobin	13.0 ± 1.8 g/dL
Platelets	288.5 ± 138.6 × 10^3^/µL
Total protein	7.1 ± 0.65 g/dL
Albumin	3.96 ± 0.50 g/dL
NSE	29.5 ± 16.7 ng/mL
CEA	6.36 ± 5.85 ng/mL
*PET–CT*	
SUVmax in the hypothalamic/pituitary region	3.47 ± 0.80
Total volume of hypothalamic/pituitary region	0.52 ± 0.13 mL
TLG of hypothalamic/pituitary region	1.33 ± 0.33 SUV × mL
SUVmax of the primary tumor	13.3 ± 3.5
volume of the primary tumor	42.0 ± 39.7 mL
TLG of the primary tumor	314.9 ± 348.0 SUV × mL
Maximum diameter of primary tumor	40.2 ± 19.0 mm
SUVmax of the lymph node metastases	11.6 ± 4.4
Volume of lymph node metastases	29.6 ± 33.0 mL
TLG of the lymph node metastases	208.6 ± 263.0 SUV × mL
Total tumor volume (primary tumor and lymph node metastases)	70.1 ± 53.0 mL
Total tumor TLG (primary tumor and lymph node metastases	512.6 ± 461.4 SUV × mL

CEA, Carcinoembryonic antigen; NSE, neuron-specific enolase; PET–CT, positron emission tomography–computed tomography; SUVmax, maximum standardized uptake value; TLG, total lesion glycolysis

### Relationship between NSE and factors

The patients were classified into two groups according to the NSE reference value (16.3 ng/mL): Six patients had NSE level below the reference value (NSE < 16.3 ng/mL) and 13 had NSE level above the reference value (NSE ≥ 16.3 ng/mL).

#### Hypothalamus/pituitary body

**Fig. 2:**
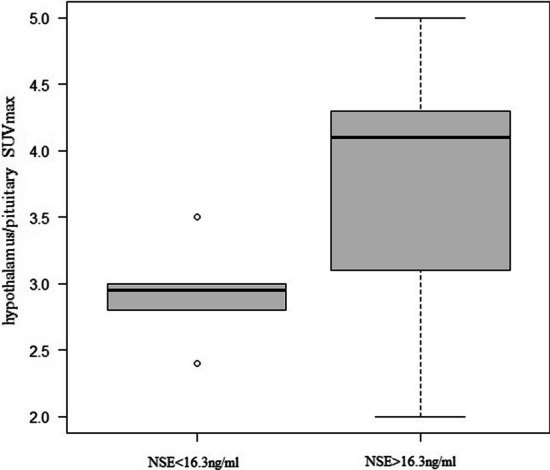
Comparison of the hypothalamus/pituitary SUVmax between the NSE < 16.3 ng/mL group and NSE > 16.3 ng/mL group

**Fig. 3:**
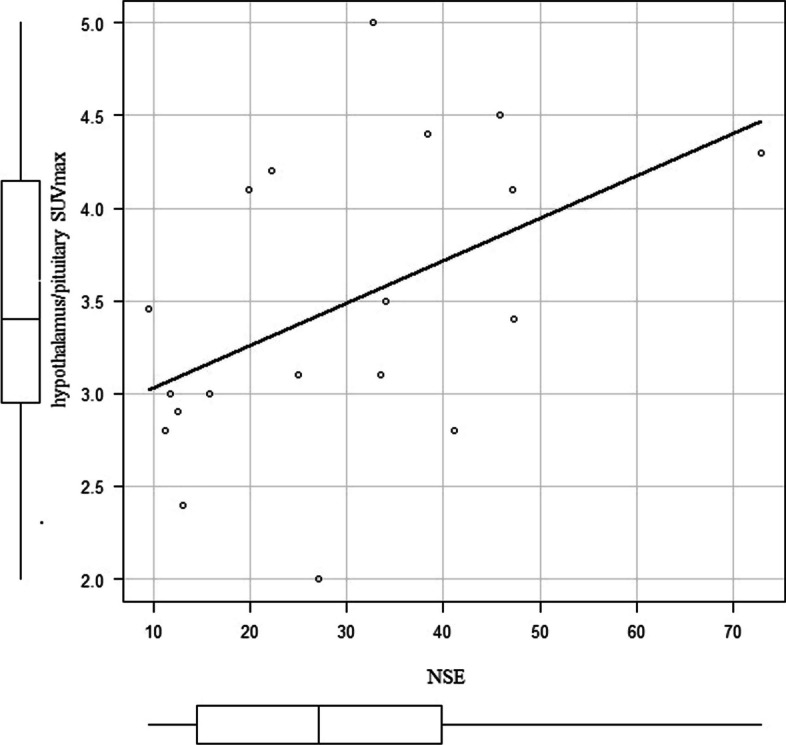
Correlation between SUVmax in the hypothalamus/pituitary and NSE level in all cases

**Fig. 4:**
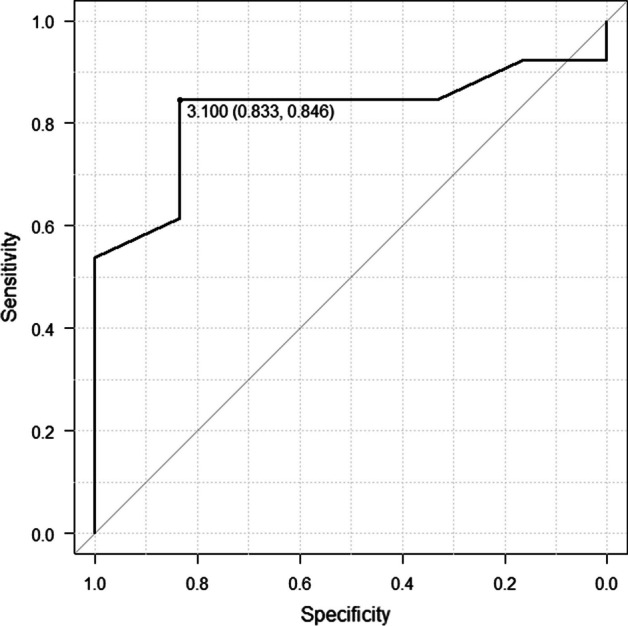
ROC analysis of hypothalamic/pituitary SUVmax and NSE below/above 16.3 ng/mL

The SUVmax of the hypothalamic/pituitary region was 2.95 in the NSE < 16.3 ng/mL group and 4.10 in the NSE ≥ 16.3 ng/mL group, and the difference was statistically significant (*p* = 0.03) (Fig. 2). A correlation coefficient of *r* = 0.458 (*p* = 0.0486) was observed between SUVmax in the hypothalamus/pituitary and NSE level (Fig. 3). ROC analysis showed that the area under the curve was 0.821, 95% confidence interval was 0.62–1, sensitivity was 0.846, and specificity was 0.833 for the NSE value (above/below the normal reference value 16.3 ng/mL) when the SUVmax of the hypothalamus/pituitary gland was 3.1 (Fig. 4).

#### Total tumor volume

Total tumor volume was 17.8 mL in the NSE < 16.3 ng/mL group and 98.9 mL in the NSE > 16.3 ng/mL group, and the difference was statistically significant (*p* = 0.03). ROC analysis revealed that the area under the curve was 0.885, 95% confidence interval was 0.727–1, sensitivity was 0.846, and specificity was 0.833 when the total tumor volume was 55.0 mL.

#### Total tumor TLG

The total tumor TLG was 126.7 SUV × mL in the NSE < 16.3 ng/mL group and 701.2 SUV × mL in the NSE > 16.3 ng/mL group, and the difference was statistically significant (*p* = 0.03). ROC analysis revealed that the area under the curve was 0.846, 95% confidence interval was 0.667–1, sensitivity was 0.615, and specificity was 1.00 for NSE value (above/below the reference value 16.3 ng/mL) when the total tumor TLG was 551.5 SUV × mL. These findings are presented in Table 2.

**Table 2:** NSE below reference value, above reference value, and comparison of factors

	NSE < 16.3 ng/mL	NSE ≥ 16.3 ng/mL	*P* value
Number of cases	6	13	
Age	73.5 years	71.0 years	0.33
Sex	Male: 5	Male: 8	0.60
	Female: 1	Female: 5	
White blood cells	7.4 × 10^3^/µL	6.8 × 10^3^/µL	0.73
Red blood cells	4.47 × 10^6^/µL	4.46 × 10^6^/µL	0.90
Hemoglobin	13.3 g/dL	13.9 g/dL	0.43
Platelets	242 × 10^3^/µL	285 × 10^3^/µL	0.28
Total protein	7.2 g/dL	7.3 g/dL	0.86
Albumin	4.2 g/dL	4.0 g/dL	0.57
CEA	2.85 ng/mL	4.80 ng/mL	0.27
NSE	12.1 ng/mL	34.1 ng/mL	< 0.01
Hypothalamus and pituitary gland SUVmax (all cases)	2.95	4.10	0.03
Total volume of hypothalamic/pituitary region	0.60 mL	0.45 mL	0.10
TLG of hypothalamic/pituitary region	1.32 SUV × mL	1.23 SUV × mL	0.63
SUVmax of the primary tumor	14.3	13.0	0.43
Volume of the primary tumor	14.9 mL	37.5 mL	0.13
TLG of the primary tumor	114.0 SUV × mL	199.0 SUV × mL	0.24
Maximum diameter of primary tumor	28 mm	49 mm	0.16
SUVmax of the lymph node metastases	9.80	13.5	0.44
Volume of lymph node metastases	3.82 mL	31.9 mL	0.04
TLG of the lymph node metastases	22.8 SUV × mL	181.4 SUV × mL	0.03
Total tumor volume of primary tumor and lymph node metastases	17.8 mL	98.9 mL	< 0.01
Total TLG of primary tumor and lymph node metastases	126.6 SUV × mL	701.2 SUV × mL	< 0.01

CEA, Carcinoembryonic antigen; NSE, neuron-specific enolase; PET–CT, positron emission tomography–computed tomography; SUVmax, maximum standardized uptake value; TLG, total lesion glycolysis

### Factors affecting the primary site

**Fig. 5:**
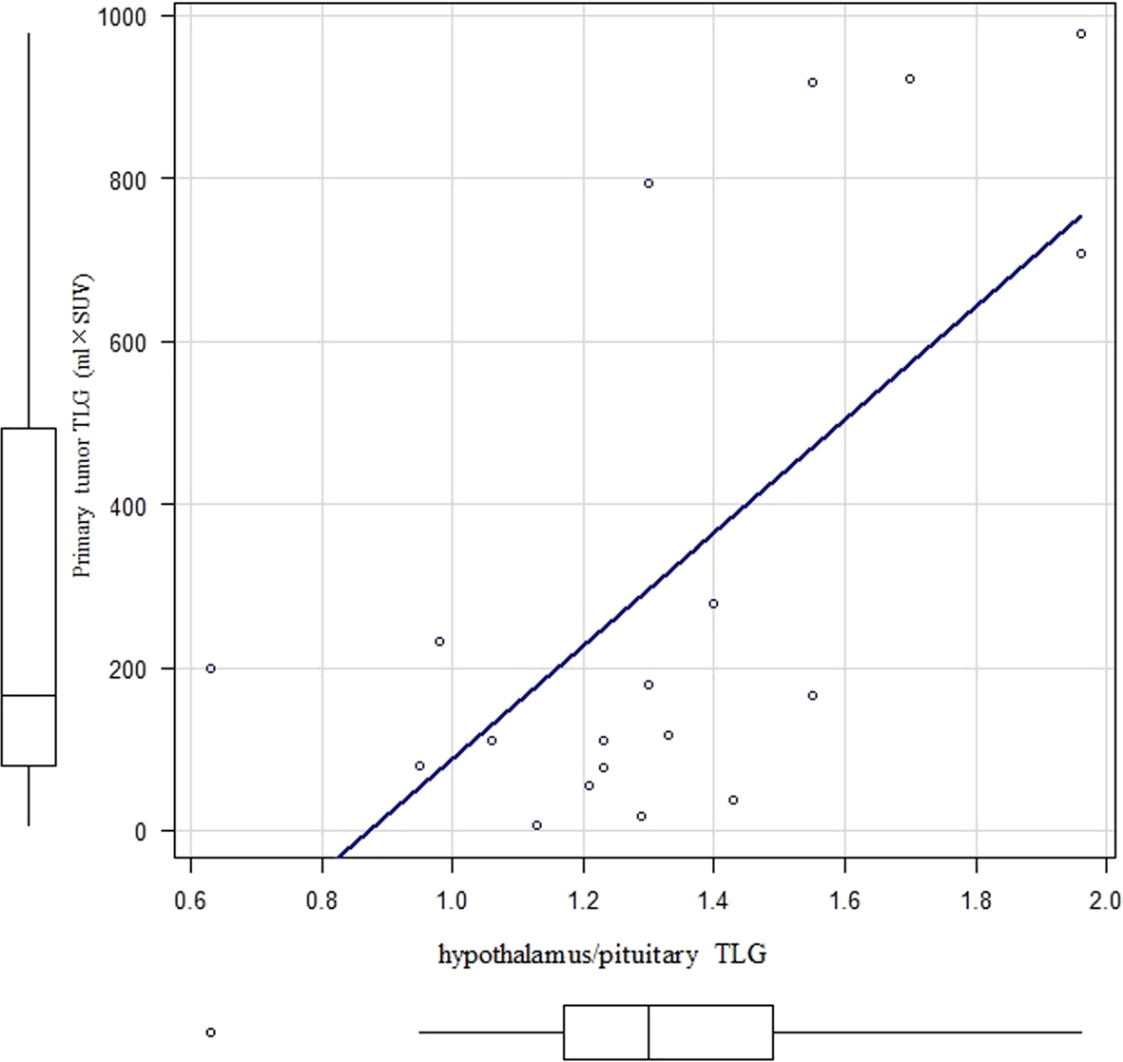
Correlation between the hypothalamus/pituitary TLG and primary tumor TLG

**Fig. 6:**
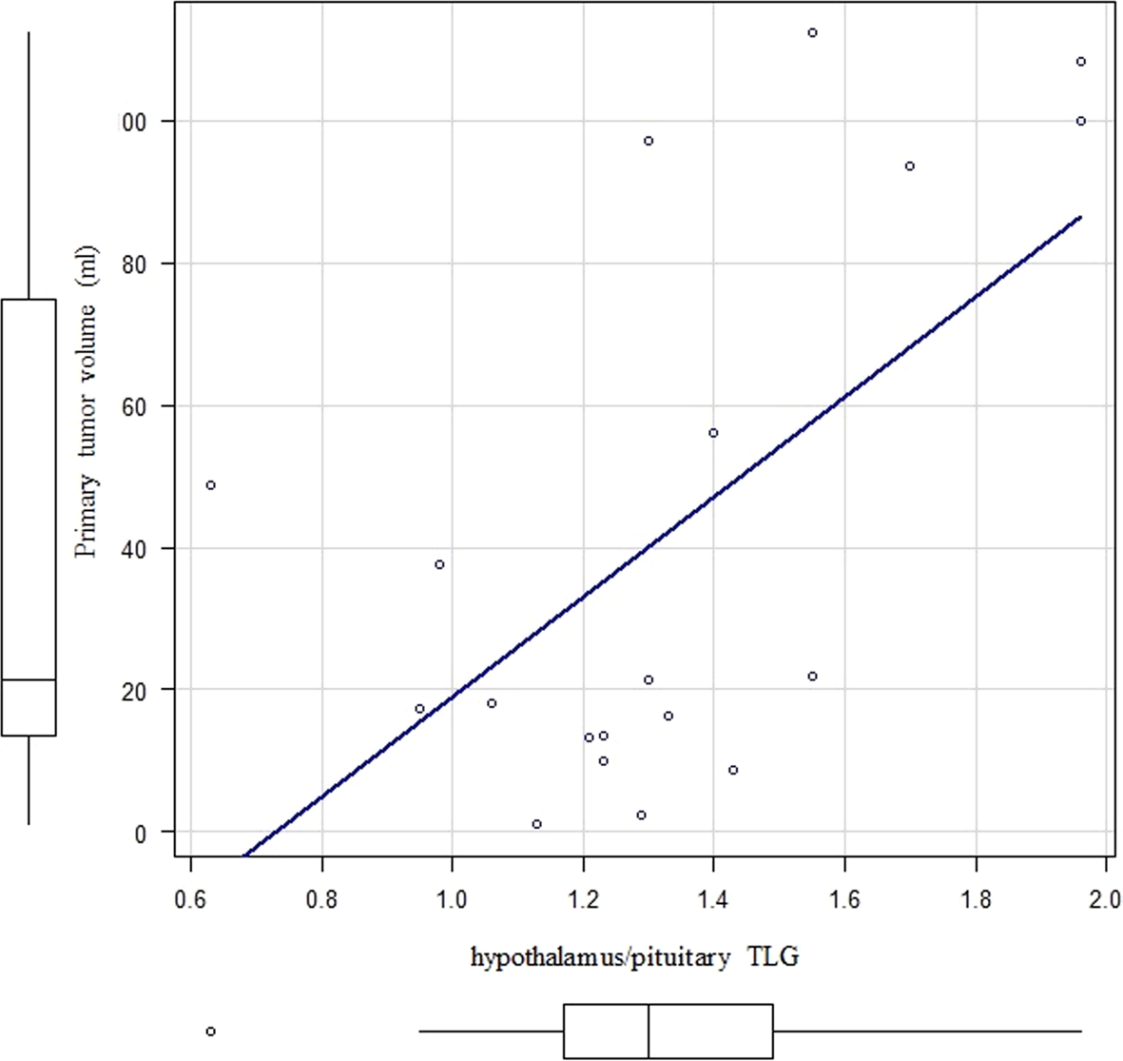
Correlation between the hypothalamus/pituitary TLG and primary tumor volume

A correlation coefficient of *r* = 0.53 (*p* < 0.01) was observed between hypothalamus/pituitary TLG and primary tumor TLG (Fig. 5). A correlation coefficient of *r* = 0.49 (*p* = 0.03) was observed between hypothalamus/pituitary TLG and primary tumor volume (Fig. 6). No statistically significant correlation was noted between hypothalamus/pituitary SUVmax/volume, NSE level, and primary tumor TLG and volume.

### Factors affecting lymph node metastases

A correlation coefficient of *r* = 0.57 (*p* < 0.01) was observed between NSE level and lymph node metastases TLG. A correlation coefficient of *r* = 0.61 (*p* < 0.01) was observed between NSE level and lymph node metastases volume. No statistically significant correlation was noted between hypothalamus/pituitary SUVmax/volume/TLG and lymph node metastases TLG and volume.

### Factors affecting the total tumor

A correlation coefficient of *r* = 0.65 (*p* < 0.01) was observed between NSE level and total tumor volume. A correlation coefficient of *r* = 0.59 (*p* < 0.01) was observed between NSE level and total tumor TLG. A correlation coefficient of *r* = 0.424 (*p* = 0.07) was observed between hypothalamus/pituitary TLG and total tumor TLG. A statistically significant correlation was noted between hypothalamus/pituitary SUVmax/volume and total tumor TLG and volume.

## Discussion

This study investigated the relationship between ^18^F-FDG uptake in the hypothalamic/pituitary, NSE level, and primary tumor in limited-stage small cell lung cancer. In this study, hypothalamic/pituitary SUVmax and NSE showed a statistically significant positive correlation. Moreover, hypothalamic/pituitary TLG and primary tumor TLG/volume showed a statistically significant positive correlation. We believe that hypothalamus and pituitary activity can affect small cell lung cancer activity.

Small cell lung cancer has been reported to exhibit endocrine abnormalities. A study of 213 cases of small cell lung cancer found that the condition in one case was complicated and that in two cases was likely complicated with Cushing's syndrome associated with ectopic adrenocorticotropic hormone (ACTH) production (Piasecka et al. 2022). Hypothyroidism and menstrual dysfunction associated with ectopic ACTH-producing tumors are reported to improve after drug treatment (Lin et al. 2009). Moreover, there is a possibility that the pituitary gland is directly involved in the syndrome of inappropriate secretion of antidiuretic hormone (SIADH). A previous study reported SIADH in 40 (11%) of 350 cases of small cell lung cancer (List et al. 1986). Pathological studies in rats and humans have reported that all pituitary adenomas stained positive for NSE to varying degrees (Noorden et al. 1984).

NSE is widely reported to be a prognostic factor for small cell lung cancer. Poor overall survival was reported in 64 cases of limited-stage small cell lung cancer, with an NSE of ≥ 46 µg/mL (Wójcik et al. 2008). A study of 523 cases of small cell lung cancer (140 limited-stage cases and 383 extensive-stage cases) reported that both limited- and extensive-stage cancers responded to initial treatment, and the survival period was favorable in the group within the NSE criteria (Zhou et al. 2017). Furthermore, a study of 301 cases of small cell lung cancer (163 limited-stage cases, 54.2%; 138 extensive-stage cases, 45.8%) found that nutritional status before treatment initiation and NSE level (15 ng/mL) were prognostic factors, in addition to the treatment strategy (localized-stage or extensive-stage chest irradiation). A previous study reported that patients with NSE > 15 ng/mL had a poor prognosis (Wang et al. 2021). A systematic review of 18 studies and 2981 cases reported that NSE is a predictor of overall survival and progression-free survival (Tian et al. 2020). However, a sub-analysis reported that NSE was not a prognostic factor in the group that underwent chemotherapy or surgery plus chemotherapy (Tian et al. 2020). NSEs have been reported to promote cancer stem cell-like properties in small cell lung cancer (Lu et al. 2022). These results suggest that NSE is a prognostic factor in the limited stage (Lu et al. 2022).

Regarding PET–CT, a study of 46 patients with limited-stage small cell lung cancer treated with chemoradiotherapy found that the median survival time was 20.6 months in the group with primary tumor SUVmax of ≥ 9.3 and 20.6 months in the group with primary tumor SUVmax of < 9.3. The median survival time was 30.9 months. The median survival time was 21 months when the SUVmax of lymph node metastases was ≥ 5.8 and 73 months when the SUVmax was < 5.8, and the difference was statistically significant (Aktan et al. 2017). However, a study of 45 cases of limited-stage small cell lung cancer reported that the SUVmax, TLG, and MTV of the primary tumor were not prognostic factors; however, the TLG and MTV of lymph node metastases were prognostic factors for survival (odds ratio: 2.6–2.8) (Jin et al. 2018). The relationship between PET–CT findings and prognosis in limited-stage small cell lung cancer remains unclear. We did not evaluate the prognosis in this study; however, we found a correlation between total tumor volume/TLG and NSE. We believe that lymph node/total tumor volume and TLG may serve as prognostic factors, in addition to NSE.

Chest chemoradiotherapy and prophylactic whole-brain radiation therapy are standard treatments for localized small cell lung cancer when the treatment response is favorable. The 5-year survival rates in 417 cases of limited-stage small cell lung cancer were 16% and 26% with conventional fractionated irradiation at 45 Gy/25 times/5 weeks and accelerated hyperfractionated irradiation at 45 Gy/30 times/3 weeks (twice a day), respectively (Turrisi et al. 1999). A study comparing conventional fractionated irradiation at 60 Gy/30 times/6 weeks and accelerated hyperfractionated irradiation at 45 Gy/30 times/3 weeks found no significant difference in survival between the two groups, and accelerated hyperfractionated irradiation remains the standard treatment (Grønberg et al. 2016).

Furthermore, prophylactic whole-brain irradiation at 25 Gy/10 doses is reported to prolong overall survival when the primary lesion is in partial or complete remission (Gregor et al. 1997). A previous study comparing three groups of prophylactic whole-brain irradiation (36 Gy/18 times, 25 Gy/10 times, and non-irradiation) found that the incidence of brain metastasis decreased in 194 cases in the irradiation group compared with the 120 cases in the non-irradiation group (hazard ratio: 0.44). Furthermore, improved survival (hazard ratio: 0.75) and overall survival (hazard ratio: 0.86) were reported in the group without brain metastasis (Péchoux et al. 2009). Therefore, prophylactic whole-brain irradiation prolongs survival. A meta-analysis of 2114 cases also reported that prophylactic whole-brain irradiation reduced the incidence of brain metastases (hazard ratio: 0.45) and prolonged survival (hazard ratio: 0.81) (Yin et al. 2019). The dose was randomized to 25 Gy/10 times (360 cases) and 36 Gy/18 times (360 cases), and the 2-year survival rate was poorer in the 36 Gy/18 dose group (42% in the 25 Gy/10 dose group and 37% in the 36 Gy/18 dose group). The incidence of brain metastasis over 2 years was reported to be 29% at 25 Gy/10 doses and 23% at 36 Gy/18 doses, with no significant difference between the values (Péchoux et al. 2009). In addition, a study of 265 patients assigned to 25 Gy/10 doses, 36 Gy/18 doses, or 36 Gy/24 doses (twice daily) reported memory impairment in the 36 Gy irradiation group (Wolfson et al. 2011). Therefore, prophylactic whole-brain irradiation has been standardized at 25 Gy/10 doses.

Whether brain metastases from small cell lung cancer are adequately radiosensitive to be controlled at low doses and have an adequate response to radiotherapy remains unknown. Several reports of whole-brain irradiation for brain metastases from small cell lung cancer exist. A study from Japan reported that the median survival time was 234 days, and the 1-year survival rate was 34.4% when brain metastases of 48 cases of small cell lung cancer were treated with whole-brain irradiation at approximately 30 Gy/10 doses. Patients with neurological symptoms associated with brain metastasis have been reported to have poor overall survival in the group with LDH above the normal range (Anami et al. 2019). The median survival of 118 days for whole-brain irradiation in 24 patients with small cell lung cancer brain metastases showed that NSE is a prognostic factor (Okada et al. 2022a). The first author's study of whole-brain irradiation in 31 patients with multiple brain metastases of non-small cell lung cancer (four or more initial cases) showed that the median survival time was 129 days (Okada et al. 2022b). In 82 cases of brain metastasis from small cell lung cancer, 33 cases of whole-brain radiotherapy plus additional stereotactic radiotherapy were compared with 49 cases of whole-brain radiotherapy alone, and the median survival times were reported to be 13.4 and 8.5 months, respectively, and improved survival rates were particularly favorable in the group with 1–3 brain metastatic lesions (Sun et al. 2018). A meta-analysis of 14,722 cases also reported that the addition of stereotactic radiotherapy improved survival (Jiang et al. 2019).

Although it is necessary to consider the status of lesions other than the brain, it is difficult to say that the treatment results of whole-brain irradiation alone for brain metastases from small cell lung cancer are satisfactory. Whether a dosage of 25 Gy/10 doses can help eliminate metastatic brain tumors, even occult micrometastatic brain lesions, remains questionable. A study of advanced stage small cell lung cancer found that the median survival time in the chemotherapy plus immunotherapy group was 12.3 months and that in the chemotherapy alone group was 10.3 months (Horn et al. 2018). Immune checkpoint inhibitors may enhance the induction of anti-tumor immunity associated with chemotherapy. However, immune checkpoint inhibitors impair endocrine organ function. Therefore, if small cell lung cancer uses the endocrine function, it may indirectly suppress the growth of small cell lung cancer through the endocrine organs. A prospective clinical study of 108 cases of non-small cell lung cancer and 66 cases of malignant melanoma found that the frequency of pituitary hypofunction in patients treated with immune checkpoint inhibitors was higher than in known reports. Furthermore, pituitary signal intensity changed on MRI. In addition, survival improved in the presence of pituitary hypofunction (Kobayashi et al. 2020). These study findings do not directly support our results; nevertheless, they provide insight into the disease mechanism.

The following inferences can be drawn from these findings: (1) In small cell lung cancer, the hypothalamus and pituitary gland are involved in tumor growth, particularly primary tumor growth (hypothalamic/pituitary TLG and primary tumor TLG/volume showed a statistically significant positive correlation); (2) NSE reflects the tumor burden, particularly lymph node metastases, and hypothalamus/ pituitary activity; and (3) prophylactic whole-brain irradiation does not eradicate microscopic brain metastases but depresses hypothalamic–pituitary regions, which reduces tumor activity. However, the endocrine function of the hypothalamic/pituitary is significantly reduced at high doses, such as 36 Gy, which may limit the therapeutic effects. Prophylactic whole-brain irradiation has been reported to cause Parkinsonian symptoms and cognitive decline in patients aged ≥ 65 years (Nakahara et al. 2015), with cognitive decline presenting 6–12 months post-radiation (Gondi et al. 2013). Prophylactic whole-brain irradiation should be reexamined from the perspective of pathophysiology.

This study has several limitations. First, the number of cases was small. Second, bias cannot be ruled out owing to the retrospective nature of the study. In addition, we only examined the limited stage in this study to unify and simplify the pathology, and the advanced stage remains a subject for future studies. We aim to conduct a prospective clinical trial after further increasing the number of cases and comparing the therapeutic effects.

## Conclusion

The study findings suggest that hypothalamic/pituitary activity and tumor activity in limited-stage small cell lung cancer are correlated.

## Data Availability

The datasets used and/or analyzed during the current study are available from the corresponding author on reasonable request.
